# Disaster risk reduction strategies for cold lahar-induced flash floods at Mount Marapi, Indonesia

**DOI:** 10.4102/jamba.v18i1.2099

**Published:** 2026-09-23

**Authors:** Mardayeli Danhas, Nurhasan Syah, Ernawati Ernawati, Eri Barlian, Iswandi Umar, Ithvi Marhamah

**Affiliations:** 1 Department of Environmental Science, State University of Padang, Padang, Indonesia; 2 Department of Geography, Faculty of Social Science, State University of Padang, Padang, Indonesia

**Keywords:** cold lahar, disaster risk reduction, multi-hazard, spatial risk assessment, Interpretive Structural Modelling

## Abstract

Cold lahar-induced flash floods represent a recurrent and complex disaster in volcanic regions, particularly in Indonesia, where interactions between volcanic activity, extreme rainfall and watershed characteristics intensified disaster risk. Despite repeated events, disaster risk reduction at the local level has remained fragmented and weakly informed by spatial risk evidence. This study aimed to analyse the impacts of the 11 May 2024 cold lahar flash flood in Agam Regency, assess spatial disaster risk potential and develop disaster risk reduction strategies to support local disaster risk governance. This study applied a mixed-methods approach, combining spatial analysis using Geographic Information Systems with multi-criteria decision-making techniques. Disaster impacts were assessed at the nagari level based on human, economic and physical indicators. Spatial risk analysis integrated flash flood hazard maps, volcanic hazard zones and land-use patterns. Interpretive Structural Modelling (ISM) was used to identify and structure disaster risk reduction strategies. The results showed that disaster impacts were highly localised along river corridors originating from both Mount Marapi and Mount Singgalang, indicating a multi-source hazard system. Spatial risk distribution was strongly influenced by land-use patterns and watershed configuration. Disaster-mitigation-based land-use control, relocation of communities in high-risk areas and the development of alternative livelihoods emerged as the main driving strategies.

**Contribution:**

The findings highlighted the importance of integrating spatial risk assessment with local planning and socio-economic interventions to reduce disaster risk. This study contributes practical insights for strengthening locally grounded and community-oriented disaster risk governance in volcanic regions of developing countries.

## Introduction

Indonesia is geologically located at the convergence of three major active tectonic plates, resulting in high levels of tectonic and volcanic activity across the archipelago (Khusna et al. 2023). Subduction processes along the Sunda Arc have produced numerous active volcanoes whose hazards extend beyond eruptive phases into the post-eruption period (Katili 1975). Volcanic materials deposited on steep slopes can be easily remobilised by intense rainfall, generating cold lava flows that frequently cause severe damage to downstream settlements, infrastructure and local livelihoods. This condition makes Indonesia one of the countries most exposed to compound volcanic and hydrometeorological hazards.

West Sumatra Province is particularly vulnerable to flash floods and lahar-related disasters due to its mountainous topography, steep watershed systems and high rainfall intensity (Badan Nasional Penanggulangan Bencana [BNPB] 2024). Between 2009 and 2019, the province experienced three volcanic eruptions (BNPB 2021). More recently, the eruption sequence of Mount Marapi, which began in December 2023 and continued throughout 2024, deposited substantial volumes of volcanic materials on surrounding slopes, creating conditions conducive to recurrent cold lahar-induced flash floods (Yanuarto 2024). Field observations and information obtained during the field investigation indicated that several localised cold lahar-induced flash flood events occurred during this eruption sequence before the major event in May 2024, which caused significant casualties and widespread damage. Within this province, Agam Regency has been among the areas most severely affected by recurrent cold lahar-induced flash floods. The repeated occurrence of cold lahar-induced flash floods has disrupted settlements, damaged infrastructure and undermined the socio-economic resilience of local communities. Such recurrent disasters pose persistent challenges for local governments and communities in managing risk and protecting livelihoods (Regional Disaster Management Agency [BPBD] Kabupaten Agam 2013).

Cold lahar-induced flash floods represent a compound hazard resulting from the interaction between extreme rainfall and unconsolidated volcanic deposits in upstream catchments. These flows transport high-energy mixtures of water, sediment and debris, often producing impacts more destructive than those of conventional flooding (Hidayat, Syah & Erianjoni 2021; Manville, Major & Fagents 2013; Pierson, Wood & Driedger 2014). Under conditions of climate change, the increasing frequency and intensity of extreme rainfall events are expected to further exacerbate the occurrence and severity of flash floods in volcanic regions, including West Sumatra. This trend highlights the growing urgency of strengthening disaster risk reduction at the local level (Intergovernmental Panel on Climate Change [IPCC] 2022).

Despite the repeated occurrence of such events, disaster risk reduction practices in West Sumatra remain constrained by the limited integration of multi-hazard processes and spatial risk information into local decision-making. Disaster impact assessments commonly focus on physical damage and economic losses, while the spatial distribution of future hazard exposure and risk remains insufficiently assessed (Putra 2024). For instance, post-event assessments typically emphasise damage inventories and casualty reports but rarely identify potential future lahar pathways, emerging risk zones or communities that may be exposed to subsequent events. This limitation is particularly critical in volcanic regions, where disaster risk is shaped by the interaction of hazard processes, land-use patterns and socio-economic conditions.

Previous studies have shown that disaster impacts are not determined solely by hazard magnitude but are also influenced by land-use decisions, institutional capacity and community vulnerability (Naheed 2021; Perez et al. 2020). Effective disaster risk reduction therefore requires area-based and locally grounded risk assessment approaches that can inform planning, relocation and livelihood strategies (Birkmann & Von Teichman 2010). However, to the best of our knowledge, no empirical study has integrated GIS-based spatial risk analysis with Interpretive Structural Modelling (ISM) to support disaster risk governance for cold lahar-induced flash floods in volcanic environments.

The cold lahar-induced flash flood event of 11 May 2024 affected several regencies and cities in West Sumatra. Agam Regency, the focus of this study, experienced extensive impacts, including 24 lives, injured 24 people and 704 displaced, hundreds of houses and dozens of infrastructure experienced damage ranging from light to severe (Yanuarto 2024). These extensive human, infrastructure and livelihood losses highlight the importance of more integrated, spatially informed disaster risk reduction planning.

Accordingly, this study aimed to analyse the impacts of the 11 May 2024 cold lahar flash flood in Agam Regency, assess spatial disaster risk potential through the integration of flash flood hazard maps, volcanic hazard zones and land-use data, and develop a structured disaster risk reduction strategy model using a multi-criteria decision-making approach. By linking spatial risk assessment with locally relevant risk reduction strategies, this study sought to provide practical insights to support local governments and communities in strengthening disaster risk governance at the nagari level. The findings are expected to contribute to more context-sensitive and actionable disaster risk reduction practices in volcanic regions of developing countries, in line with the principles of the Sendai Framework for Disaster Risk Reduction (Naheed 2021).

## Research methods and design

### Study design

This study adopted a quantitative spatial research design to assess the impacts of cold lahar-induced flash floods and spatial disaster risk in Agam Regency. Geographic Information System (GIS)-based spatial analysis was employed to map disaster impacts and assess disaster risk through the integration of flash flood hazard maps, volcanic hazard zones and land-use data. The spatial analysis was subsequently integrated with ISM to develop disaster risk reduction strategies relevant to local disaster governance. The combination of GIS and ISM was chosen because it enables spatial identification of disaster risk while providing a structured decision-support framework for developing locally appropriate disaster risk reduction strategies.

### Study area

The study area comprised river catchments originating from Mount Marapi and traversing several nagari in Agam Regency, West Sumatra Province, Indonesia. These areas were directly affected by the cold lahar-induced flash flood event of 11 May 2024. The region is characterised by steep terrain, abundant unconsolidated volcanic deposits and dense settlements along river corridors, making it highly vulnerable to cold lahar-induced flash floods.

### Data source and collection

This study utilised both primary and secondary data sources. Secondary data consisted of spatial datasets and disaster impact records obtained from official reports of Agam Regency Disaster Management Agency (BPBD) and relevant sectoral agencies. These data included information on human losses, economic damage, physical infrastructure damage, hazard maps, land use data and administrative boundaries.

Primary data were collected through field observations, on-site verification of affected locations and semi-structured interviews with local stakeholders. These activities were conducted to validate secondary data and ensure consistency between field conditions and the results of spatial analysis. Geographic Information Systems were used as the main analytical platform for data processing and spatial analysis.

### Disaster impact assessment

Disaster impacts were assessed at the nagari level and classified into three main categories: human, economic and physical impacts. This classification was used to structure the assessment in a comparable manner across affected areas (Table 1).

**TABLE 1:** Classification of disaster impacts.

Classification	Parameters	Data source
Human impact (40%)	Fatalities Displaced population	Agam Regency Disaster Management Agency (BPBD)
Economy impact (30%)	Agriculture land (paddy fields) Plantation areas Fisheries	Department of Agriculture; Department of Marine Affairs and Fisheries, Agam Regency
Physical impact (30%)	Housing Irrigation systems Roads and bridges Clean water infrastructure	Department of Public Works, Agam Regency

*Source*: BPBD Kabupaten Agam, 2013, *Kajian Risiko Bencana Kabupaten Agam* [*Agam regency disaster risk study*], viewed 28 August 2026, from https://dkms.bnpb.go.id/api/backend/uploads/documents/1777544716_Kajian%20Risiko%20Bencana%20Kabupaten%20Agam%20Sumatera%20Barat%20Tahun%20%202014-2018.pdf

Human impacts included fatalities and displaced populations. Economic impacts comprised damage to agricultural land, plantation areas and fisheries. Physical impacts covered damage to housing, irrigation systems, roads and bridges and clean water infrastructure. The weighting scheme was adopted from the Indonesian National Disaster Management Agency (BNPB) Guidelines for Disaster Risk Assessment (*Peraturan Kepala Badan Nasional Penanggulangan Bencana Nomor 2 Tahun 2012 Tentang Pedoman Umum Pengkajian Risiko 2012*), which prioritises human impacts while also accounting for economic and physical losses. Accordingly, human impacts were assigned a weight of 40%, while economic and physical impacts were each assigned weights of 30%.

For each impact variable, data were normalised to a range of 0–1 using the min–max normalisation method to enable comparison across nagari. A composite disaster impact index was then calculated by aggregating the weighted human, economic and physical impact components. Based on the resulting index values, nagari were classified into low, moderate and high impact categories as presented in Table 2.

**TABLE 2:** Disaster impact categories.

Index (X’_i_)	Category
0 < Xi < 0.3	Low
0.3 < Xi < 0.6	Moderate
0.6 < Xi < 1	High

*Source*: Umar, I. & Triyatno, 2024, ‘Flood hazard mitigation at Tarusan watershed, south Pesisir District, West Sumatera province’, *Jurnal Pengelolaan Sumberdaya Alam Dan Lingkungan* 14(1), 101–108. https://doi.org/10.29244/jpsl.14.1.101-108

### Disaster risk spatial analysis

Spatial disaster risk analysis was conducted to identify areas with high potential losses from cold lahar-induced flash floods. The analysis integrated hazard and vulnerability components using an overlay technique in a GIS environment. The spatial datasets used in this study are summarised in Table 3. These datasets were selected to represent hazard components and vulnerability factors relevant to cold lava flash flood risk in the Mount Marapi area.

**TABLE 3:** Spatial data used in the study.

Spatial data	Description	Source
Flash flood hazard (H1)	Identification of flash flood hazard classes based on: • Topography • Distribution of flood-induced sediment deposits • Historical flash flood records • River conditions	Kementerian Pekerjaan Umum (2012)
Volcanic hazard (H2)	Identification of volcanic hazard classes based on: • Pyroclastic fall • Lava flow zones • Lahar flow zones	BNPB (2019)
Land use pattern (V)	Basis for analysing disaster impact risk on human, physical and economic components	Geospatial Information Agency (BIG)
Administrative boundaries	Basis for disaster impact analysis at the nagari level	Geospatial Information Agency (BIG)

Note: Please see the full reference list of this article, Danhas, M., Syah, N., Ernawati, E., Barlian, E., Umar, I. & Marhamah, I., 2026, ‘Disaster risk reduction strategies for cold lahar-induced flash floods at Mount Marapi, Indonesia’, *Jàmbá: Journal of Disaster Risk Studies* 18(1), a2099. https://doi.org/10.4102/jamba.v18i1.2099, for more information.

BNPB, Badan Nasional Penanggulangan Bencana.

Disaster risk was estimated by combining flash flood hazard, volcanic hazard and land-use vulnerability into a composite risk index. The resulting risk values were normalised and classified into low, moderate and high-risk categories, following the same classification scheme used for disaster impact assessment.

### Development of disaster risk reduction

The results of the spatial impact and risk analyses were interpreted descriptively to identify spatial patterns and underlying drivers of cold lahar flash flood risk. Interpretive Structural Modelling was employed to develop a structured disaster risk reduction strategy framework at the nagari level.

The ISM process involved decomposing disaster risk reduction strategies into key elements and sub-elements, followed by the identification of contextual relationships among the sub-elements through expert judgement. The expert panel consisted of five purposively selected participants representing academia, government agencies and local communities. In ISM, expert selection emphasises participants’ knowledge, experience and decision-making roles rather than sample size, as the method aims to capture structural relationships among key elements rather than produce statistically generalisable findings. However, the relatively small number of experts may limit the generalisability of the proposed strategy model beyond the study area. Therefore, the findings should be interpreted as a context-specific decision-support framework for Agam Regency, and future studies are encouraged to involve a larger and more diverse panel of experts to further validate and refine the proposed strategy model.

Based on pairwise comparisons, a Structural Self-Interaction Matrix (SSIM) was constructed and converted into a Reachability Matrix. Transitivity rules were applied iteratively to establish a stable hierarchical structure. The resulting structure was analysed using a Driver Power–Dependence matrix, where driver power represents the extent to which a sub-element influences other sub-elements, while dependence indicates the degree to which a sub-element is influenced by others (Thakkar 2021; Warfield 1976). Based on these measures, the sub-elements were classified into four categories: autonomous, dependent, linkage and independent. This analysis enabled the identification of key driving strategies for disaster risk reduction in lahar-prone areas.

### Ethical considerations

This study involved expert consultations conducted with informed consent. No personal, confidential or sensitive information was collected from participants, and the data were used solely for research purposes. Ethical clearance to conduct this study was obtained from the Institute for Research and Community Service of the Universitas Negeri Padang (Ref. No. 835/UN35.15/TU/2026).

## Results

### Impact assessment

#### Spatial distribution of disaster impacts

The cold lahar flash flood event of 11 May 2024 affected six sub-districts within the Mount Marapi area of Agam Regency, namely Ampek Angkek, Ampek Koto, Candung, Sungaipua, Baso and Banuhampu. These sub-districts comprise a total of 35 nagari. The spatial distribution of disaster impacts at the nagari level is presented in Figure 1.

**FIGURE 1:**
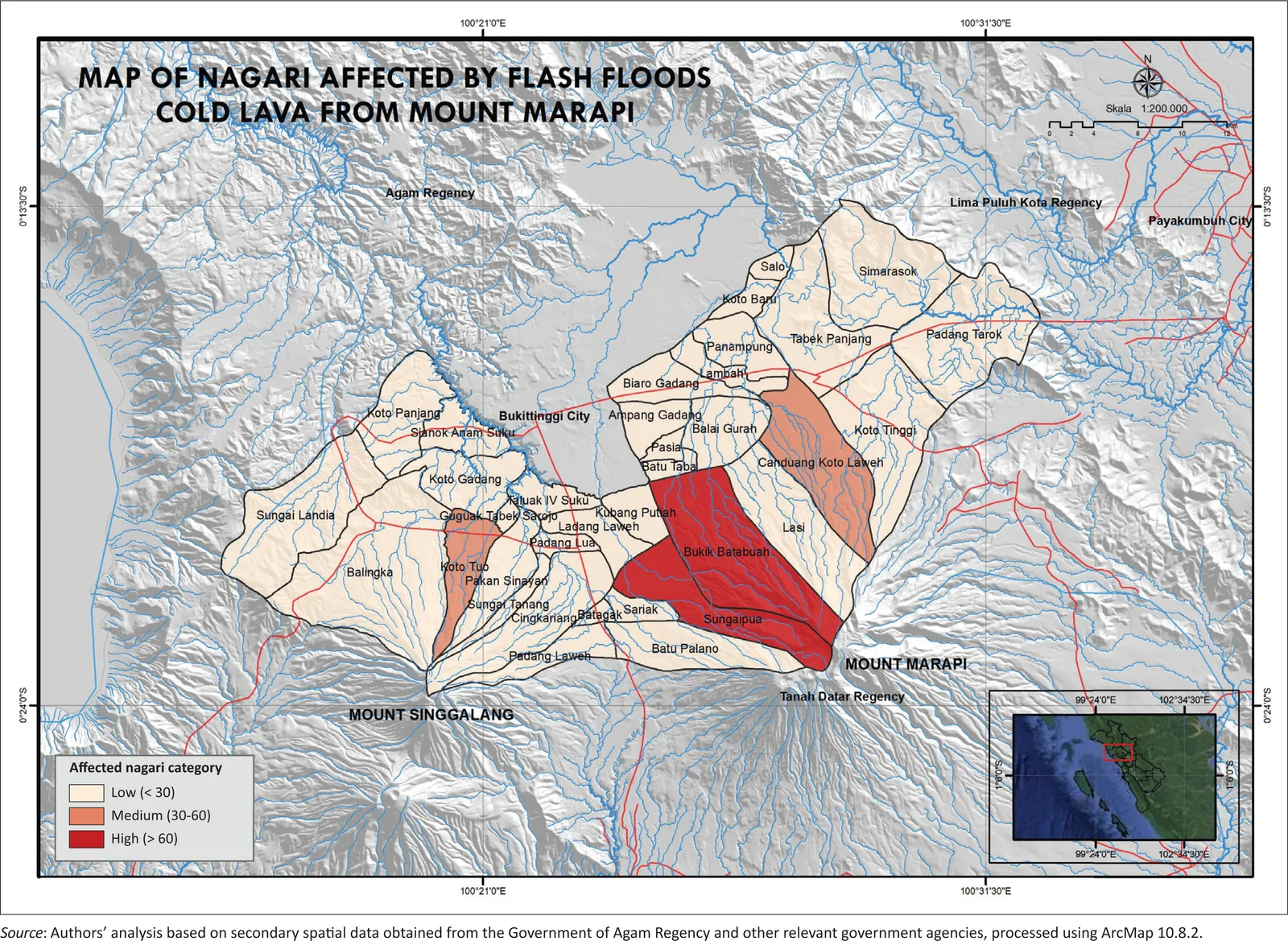
Spatial distribution of nagari affected by cold lahar flash floods from Mount Marapi, Agam Regency.

Figure 1 shows that moderate to high disaster impacts were spatially concentrated along river corridors originating from the slopes of Mount Marapi. The most severely affected nagari were Bukik Batabuah and Sungaipua, while Candung Koto Laweh experienced moderate impacts. Importantly, impacted areas were not limited to nagari located directly on the slopes of Mount Marapi.

Several affected nagari were situated within watersheds originating from the slopes of Mount Singgalang, particularly Koto Tuo, which experienced moderate impacts. This pattern indicates that the 11 May 2024 flash flood event resulted from the interaction between post-eruption volcanic conditions at Mount Marapi and widespread extreme rainfall affecting multiple upstream catchments across two adjacent mountain systems.

#### A multi-source hazard analysis

The disaster impact map further reveals that flash flood flows originated from multiple sub-watersheds draining both Mount Marapi and Mount Singgalang. On the slopes of Mount Marapi, flash flood processes were dominated by cold lahar flows generated by the remobilisation of unconsolidated volcanic deposits. In contrast, flash floods on the slopes of Mount Singgalang were primarily driven by intense rainfall, producing debris flows and rapid surface runoff within steep watersheds with limited infiltration capacity.

Overall, affected nagari were aligned along river corridors originating from both mountains, with higher impact concentrations observed in river confluence zones and downstream areas. Although most nagari (31 out of 35) were classified as low-impact areas based on the composite impact index, the presence of nagari with moderate to high impacts on both mountain slopes demonstrates that the hazard sources were multi-origin rather than singular.

Nagari experiencing more severe impacts were predominantly located in areas exposed to cold lahar flows originating from Mount Marapi. In these locations, cold lahar flash floods caused extensive damage to settlements, transportation infrastructure, water supply systems and productive land uses, including agriculture, plantations and fisheries. Social impacts, including fatalities and disruptions to local economic activities, were also more pronounced in these high-exposure areas.

### Risk analysis

#### Spatial distribution of disaster risk

Disaster risk potential was assessed by integrating flash flood hazard maps, volcanic hazard maps and land-use patterns to identify spatial variations in cold lahar flash flood risk across Agam Regency. This integrated approach enabled a more comprehensive understanding of how interactions between multiple hazard sources and land-use intensity influence potential future losses. The spatial distribution of disaster risk is presented in Figure 2.

**FIGURE 2:**
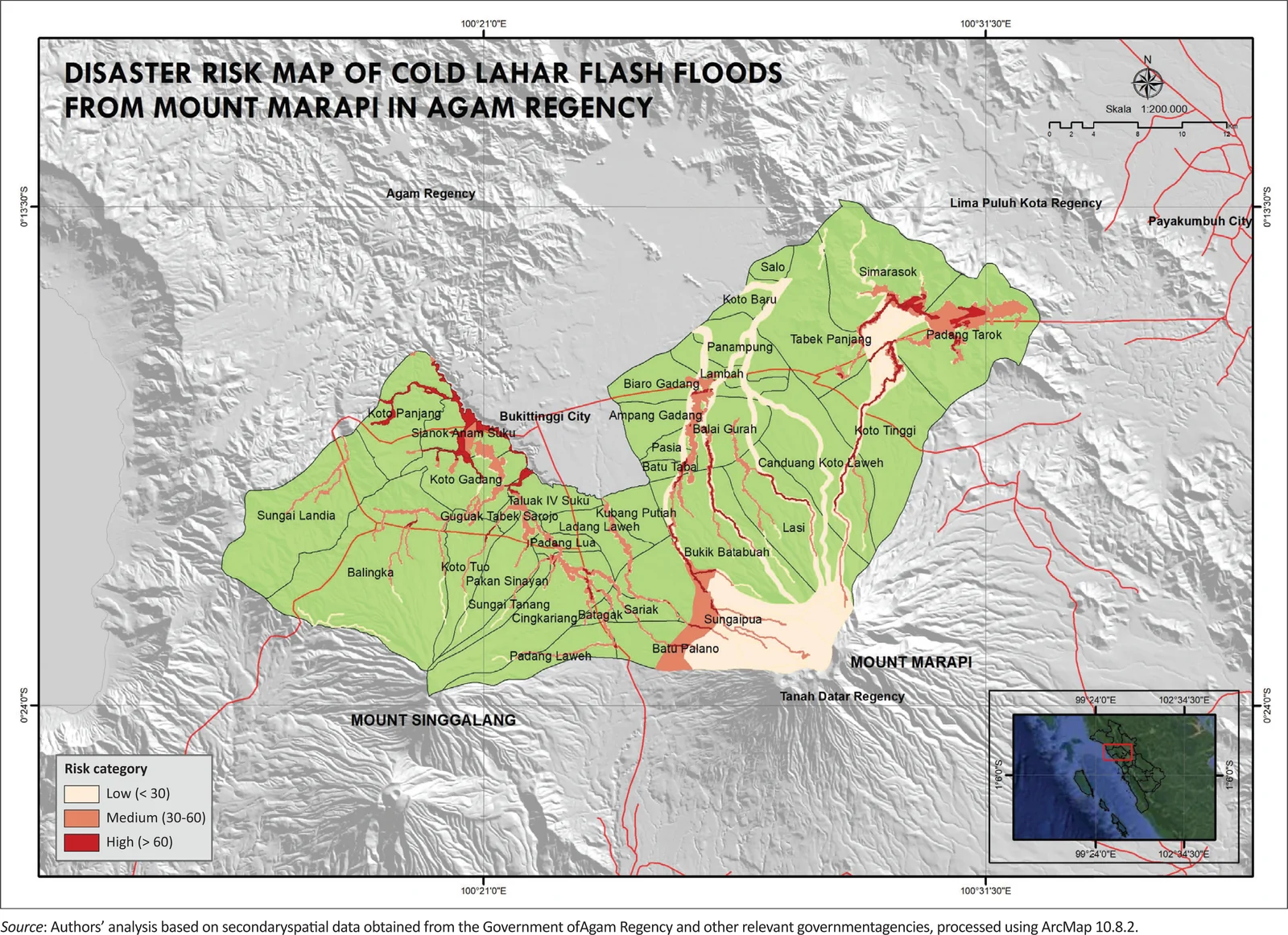
Disaster risk map of cold lahar flash floods from Mount Marapi in Agam Regency.

Figure 2 indicates that high-risk zones were predominantly associated with nagari characterised by dense settlement areas and intensive land use. In these areas, flash floods posed elevated risks to human safety, infrastructure and economic activities, as observed in Sianok Anam Suku and Padang Tarok Nagari. Conversely, areas dominated by primary and secondary forests generally exhibited lower risk levels, reflecting lower exposure and reduced concentrations of vulnerable assets.

#### Priority nagari for disaster risk reduction

Accordingly, the spatial risk analysis identified several priority nagari where disaster risk reduction efforts should be concentrated. Table 4 presents the five nagari with the largest high-risk areas, representing the highest priorities for targeted disaster risk reduction interventions in Agam Regency.

**TABLE 4:** Nagari with the largest high-risk areas in Agam Regency.

Nagari	Sub-district	Area (m^2^)			
		High	Moderate	Low	Total
Bukik Batabuah	Canduang	286,030	1,316,238	5,885,589	7,487,857
Padang Tarok	Baso	603,793	2,667,817	566,611	3,838,221
Simarasok	Baso	491,744	752,973	1,705,693	2,950,410
Sianok VI Suku	IV Koto	864,387	720,977	32,555	1,617,919
Koto Panjang	IV Koto	519,207	125,050	21,400	665,657

The analysis shows that the five nagari with the largest high-risk areas represent the primary priorities for disaster risk reduction interventions in Agam Regency. Although not among these five, Nagari Sungaipua exhibited one of the largest combined moderate- and high-risk zones. Together with Bukik Batabuah, Sungaipua is located along major river corridors draining the slopes of Mount Marapi, making both nagari highly susceptible to recurrent cold lahar-induced flash floods. This finding suggests that disaster risk reduction priorities should consider not only the spatial extent of high-risk areas but also the continuity of moderate-risk zones, which may transition into higher-risk areas during extreme rainfall events.

The integrated risk map further indicates that Mount Marapi, an active volcano, and Mount Singgalang, an inactive volcano, form an interconnected watershed system influencing the spatial distribution of disaster risk. Upstream areas on both mountain slopes function as hazard source zones characterised by volcanic deposits and steep terrain, whereas middle and downstream watershed areas, where land-use intensity and population density are higher, act as zones of risk accumulation

These findings demonstrate that disaster risk in Agam Regency is not determined solely by natural hazard intensity but is strongly influenced by land-use patterns and the spatial concentration of human activities. Nagari located along river confluences and downstream watershed corridors experienced higher levels of risk because of the combined effects of hazard convergence and greater exposure.

Overall, the integrated spatial analysis confirms that cold lahar flash flood risk in Agam Regency is dynamic and multidimensional. The combination of flash flood hazards, volcanic hazards and land-use characteristics proved effective in identifying priority nagari requiring targeted disaster risk reduction interventions. These results provide an important empirical basis for developing area-based disaster risk reduction strategies and for strengthening local disaster risk governance in volcanic regions.

### Disaster risk reduction strategy

Based on the results of the disaster impact and spatial risk analyses, disaster risk reduction strategies for cold lahar flash floods in Agam Regency were formulated through a Focus Group Discussion involving key stakeholders. The discussion included representatives from local government agencies, academics and community leaders, ensuring that the proposed strategies reflected both technical considerations and local socio-institutional contexts. Seven disaster risk reduction strategy sub-elements were identified, as summarised in Table 5.

**TABLE 5:** Disaster risk reduction strategy elements.

Element	Strategy
E1	Relocation of communities living in high-risk areas
E2	Strengthening early warning systems and emergency preparedness
E3	Development of alternative livelihoods for local communities
E4	Structural mitigation through check dams
E5	Capacity building of nagari communities
E6	Disaster-mitigation-based land-use control
E7	Reforestation of slopes and riverbanks

*Source*: Focus Group Discussion Result Analysis, 2025

The identified strategies encompassed both structural and non-structural measures, addressing physical, social and governance dimensions of disaster risk reduction. These included community relocation from high-risk zones, strengthening early warning systems and emergency preparedness, development of alternative livelihoods, structural mitigation through check dams, capacity building at the nagari level, disaster-mitigation-based land-use control and reforestation of slopes and riverbanks.

Interpretive Structural Modelling was applied to analyse the interrelationships among the strategy sub-elements and to identify their relative roles within the disaster risk reduction system. The Driver Power–Dependence analysis revealed that three sub-elements had high driver power and low dependence, indicating their strategic importance in influencing other risk reduction measures. These key driving strategies were disaster-mitigation-based land-use control (E6), relocation of communities living in high-risk areas (E1) and development of alternative livelihoods for local communities (E3), as illustrated in Figure 3.

**FIGURE 3:**
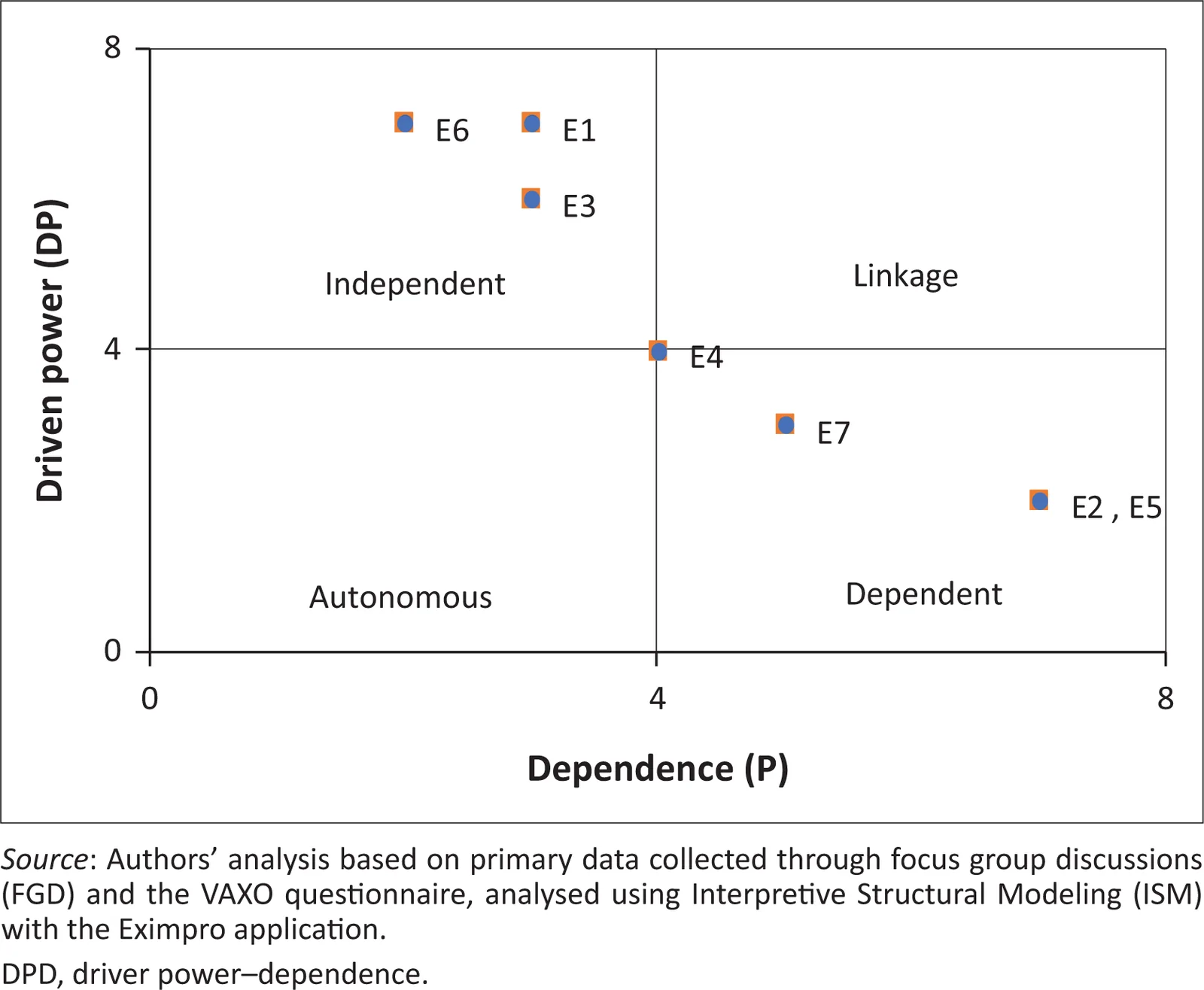
Driver power–dependence matrix of disaster risk reduction strategies for cold lahar flash floods from Mount Marapi in Agam Regency.

Based on the ISM results, the disaster risk reduction strategies were structured into four hierarchical levels, as shown in Figure 4.

**FIGURE 4:**
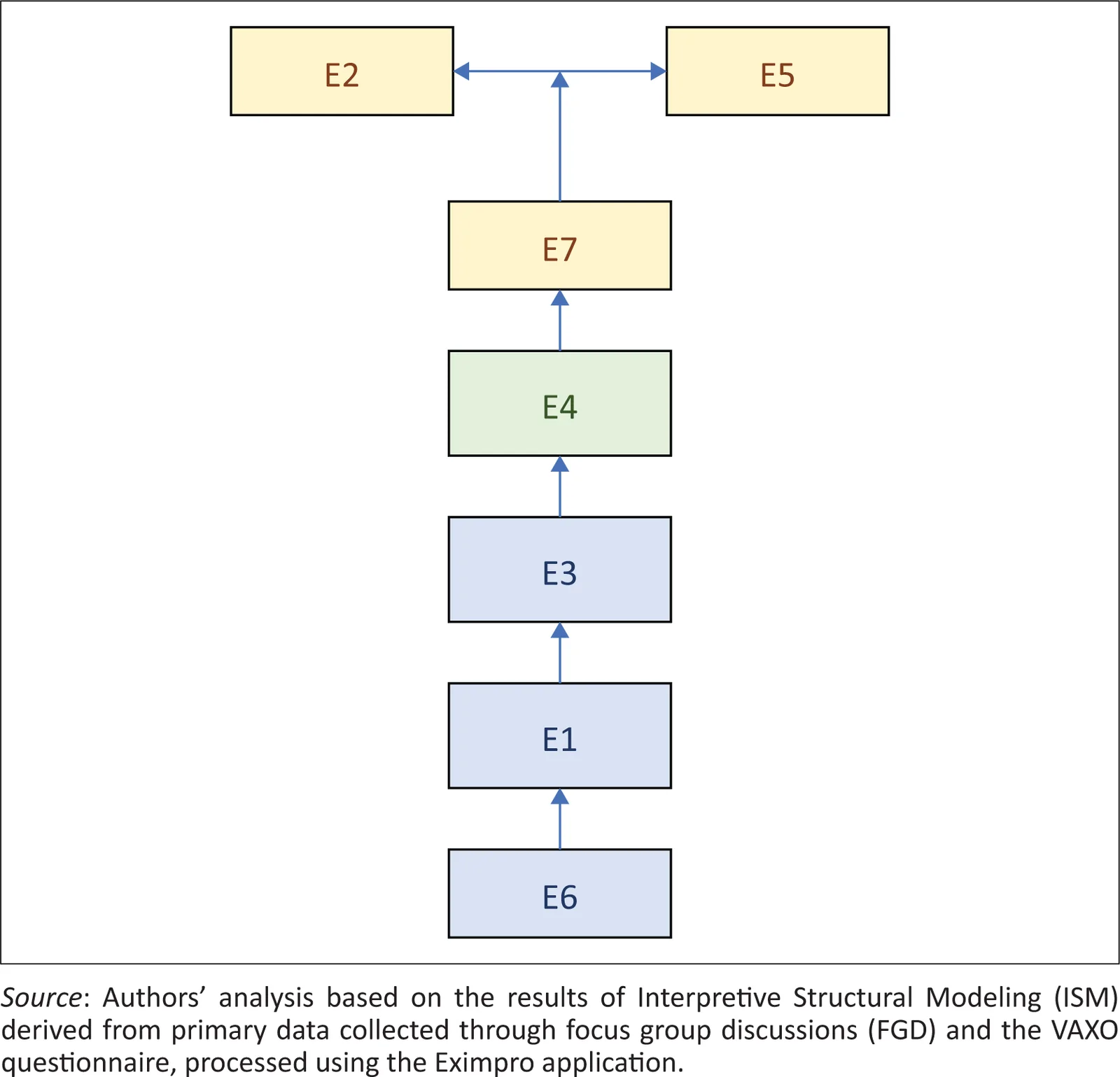
Hierarchical structure of disaster risk reduction strategies for cold lahar flash floods from Mount Marapi in Agam Regency.

At the highest priority level were disaster-mitigation-based land-use control and the relocation of communities from high-risk areas. These strategies function as foundational interventions, as they directly reduce exposure and vulnerability in areas most affected by cold lahar flash floods. The development of alternative livelihoods emerged as a complementary priority, supporting long-term risk reduction by reducing economic dependence on hazard-prone land and enhancing community resilience.

Other strategies, including strengthening early warning systems, capacity building at the nagari level, structural mitigation through check dams and reforestation, occupied intermediate levels within the hierarchical structure. While these measures are important, the ISM results indicate that their effectiveness is strongly influenced by the successful implementation of land-use control, relocation and livelihood diversification.

Overall, the structured strategy framework highlights the central role of spatial planning and socio-economic interventions in reducing cold lahar-induced flash flood risk in Agam Regency. The findings emphasise that effective disaster risk reduction in volcanic regions requires integrated, area-based governance approaches that align land-use regulation, community relocation and livelihood strategies within a coherent policy framework.

Nevertheless, the proposed strategy hierarchy should be interpreted within the context of the expert panel involved in this study. As ISM relies on expert judgement to establish contextual relationships among strategy elements, the resulting hierarchical structure reflects the collective judgements of the five participating experts. Consequently, different expert panels with different disciplinary backgrounds, institutional perspectives or local experiences may generate somewhat different hierarchical structures and strategic priorities.

## Discussion

This study demonstrates that cold lahar-induced flash floods in the Mount Marapi region constitute a multi-source and multidimensional disaster system in which disaster risk is generated through the interaction of volcanic processes, hydrometeorological conditions, watershed configuration and land-use patterns. The finding that affected nagari were located not only on the slopes of Mount Marapi but also within watersheds influenced by the inactive Mount Singgalang indicates that disaster impacts cannot be explained solely by volcanic activity. Instead, the spatial distribution of impacts reflects interconnected upstream catchments that simultaneously contribute runoff and sediment during extreme rainfall events. Similar processes have been documented in other volcanic regions, particularly at Mount Merapi, where rainfall-triggered lahars continued to generate destructive floods for several years after the 2010 eruption as unconsolidated volcanic deposits were repeatedly remobilised during intense rainfall events. These observations suggest that, as in the case of Mount Merapi, cold lahar hazards associated with Mount Marapi are likely to persist long after eruptive activity has declined, highlighting the need for long-term watershed management rather than short-term emergency response alone (Andreastuti et al. 2023; De Bélizal et al. 2013).

Beyond documenting disaster impacts, the integrated spatial analysis revealed the added value of combining flash flood hazards, volcanic hazard zones and land-use information to identify areas with elevated future disaster risk. The spatial risk analysis showed that several nagari with limited impacts during the 11 May 2024 event nevertheless exhibited high levels of potential future risk because of the convergence of hazardous river corridors, volcanic processes and intensive land use. This result highlights the importance of spatially explicit risk assessment as a proactive decision-support tool that enables local governments to identify priority intervention areas before disasters occur, consistent with the Sendai Framework for Disaster Risk Reduction, which emphasises disaster risk understanding as the basis for prevention, preparedness and resilience building (United Nations Office for Disaster Risk Reduction [UNDRR] 2015).

The principal contribution of this study lies in linking spatial disaster risk assessment with ISM to translate spatial evidence into strategic policy priorities. While spatial analysis identifies where disaster risk is concentrated, the ISM approach explains which interventions should be prioritised and how they interact within a governance system. Previous studies have predominantly examined either hazard mapping or disaster management strategies separately (Terzi et al. 2019; Thouret et al. 2020), whereas the present study integrates both approaches into a structured decision-support framework for local disaster risk governance. This integration provides a practical basis for prioritising interventions at the nagari level and supports more evidence-based disaster risk reduction planning in volcanic environments.

The ISM results indicate that disaster risk reduction strategies form a hierarchical governance system in which land-use control functions as the principal driving factor influencing the effectiveness of subsequent interventions. As the strategy with the highest driver power, disaster-mitigation-based land-use regulation provides the institutional foundation for controlling settlement expansion, protecting river corridors and limiting development in hazard-prone areas. This finding reinforces previous studies emphasising that disaster risk reduction is fundamentally a governance challenge in which spatial planning plays a central role in reducing exposure and increasing long-term resilience (Birkmann & Von Teichman 2010; Perez et al. 2020). Community relocation emerged as the next strategic priority but should not be regarded as a purely technical intervention. Its long-term success depends on complementary socio-economic measures, particularly the development of alternative livelihoods that reduce economic vulnerability and discourage the reoccupation of hazardous areas. Nevertheless, relocation remains politically and socially sensitive, especially in rural volcanic communities where livelihoods, land tenure and social attachment strongly influence relocation decisions. Future research is needed to examine community willingness to relocate together with the adequacy of compensation mechanisms and post-relocation livelihood sustainability.

Structural measures, including check dams, together with environmental interventions such as slope and riverbank reforestation, occupied intermediate positions within the ISM hierarchy, indicating that their effectiveness depends on broader governance conditions rather than engineering performance alone. From a policy perspective, structural mitigation such as check dams should therefore be prioritised in nagari where land-use regulations and watershed conservation programmes have already been implemented (Birkmann & Von Teichman 2010; UNDRR 2015). Otherwise, uncontrolled land-use change will continue to generate excessive sediment loads, leading to rapid sediment accumulation within check dams, increasing maintenance requirements and reducing their long-term effectiveness. This finding supports integrated and place-based disaster risk reduction approaches in which structural and non-structural measures complement one another within a comprehensive watershed management framework.

Strategies related to early warning systems, emergency preparedness, and capacity building exhibited relatively high levels of dependence within the ISM hierarchy, indicating that these measures are most effective when supported by upstream interventions addressing land-use management, spatial planning and socio-economic resilience. The resulting strategy hierarchy therefore suggests that disaster risk reduction for cold lahar-induced flash floods should prioritise preventive and transformative governance interventions while maintaining preparedness and emergency response as complementary components of an integrated disaster management system. Although the proposed hierarchy provides a structured representation of disaster risk reduction priorities for Agam Regency, it reflects the collective judgements of the five experts involved in the ISM process. Different expert panels with different disciplinary backgrounds or institutional perspectives may therefore generate somewhat different strategic hierarchies. Consequently, the proposed framework should be interpreted as a context-specific decision-support model that can be further validated and refined through future studies involving larger and more diverse expert panels.

### Strengths and limitations

A key strength of this study lies in its integration of GIS-based spatial risk analysis with ISM, enabling the translation of spatial risk evidence into a structured decision-support framework for disaster risk reduction. By combining hazard mapping with strategy prioritisation, the proposed framework provides practical guidance for local governments in identifying priority intervention areas and selecting context-appropriate risk reduction measures.

Nevertheless, several limitations should be acknowledged. Firstly, the ISM analysis was based on the judgements of a purposively selected panel of five experts. Although ISM emphasises expert knowledge rather than statistical representativeness, a larger and more diverse expert panel may generate different structural relationships and strategic priorities. Secondly, the spatial analysis relied primarily on secondary datasets, including hazard maps and land-use information, whose accuracy and temporal resolution may influence the precision of the resulting risk assessment. Thirdly, the analysis focused on the major cold lahar-induced flash flood event of 11 May 2024. Consequently, the proposed risk patterns and strategy framework may not fully represent future events occurring under different climatic, volcanic or watershed conditions. Finally, vulnerability was represented mainly through land-use characteristics and did not incorporate primary community-level vulnerability data, such as household socio-economic conditions, risk perception, coping capacity or adaptive behaviour.

Future research should therefore integrate multi-temporal hazard data, community-based vulnerability surveys and scenario-based modelling to improve the robustness of spatial risk estimation and to further validate and refine the proposed disaster risk reduction strategy framework.

## Conclusion

Based on the findings of this study, the following conclusions can be drawn:

- Cold lahar-induced flash flood risk in Agam Regency is generated by the interaction of volcanic processes, extreme rainfall, watershed configuration and land-use patterns rather than by volcanic activity alone. The integration of GIS-based spatial risk analysis revealed that high-risk areas extend beyond the slopes of Mount Marapi into interconnected watersheds, demonstrating the importance of multi-source hazard assessment for identifying priority areas for disaster risk reduction.
- By integrating spatial risk analysis with ISM, this study provides a structured decision-support framework that translates spatial risk evidence into strategic priorities for local disaster risk governance. The ISM results identify disaster-mitigation-based land-use control as the principal driving strategy, followed by community relocation and livelihood development, while structural mitigation and emergency preparedness function as complementary interventions within an integrated disaster risk reduction system.
- The ISM results indicate that effective disaster risk reduction requires a hierarchical and integrated approach. Accordingly, for Agam Regency, these findings imply that revising and enforcing disaster-sensitive spatial zoning regulations should be prioritised before expanding investments in additional early warning infrastructure. Strengthening land-use governance, watershed management and community resilience is likely to provide greater long-term reductions in disaster risk than relying primarily on emergency response measures. The proposed framework offers practical guidance for local governments in developing evidence-based, spatially informed disaster risk reduction strategies for volcanic regions.
